# Outcome of postoperative critically ill patients with heparin-induced thrombocytopenia: an observational retrospective case-control study

**DOI:** 10.1186/cc5100

**Published:** 2006-11-16

**Authors:** Elise M Gettings, Kathryn A Brush, Elizabeth M Van Cott, William E Hurford

**Affiliations:** 1 Department of Anesthesia and Critical Care, Massachusetts General Hospital, 55 Fruit Street, Boston, MA 02114, USA; 2 Department of Nursing, Massachusetts General Hospital, 55 Fruit Street, Boston, MA 02114, USA; 3 Department of Clinical Pathology, Massachusetts General Hospital, 55 Fruit Street, Boston, MA 02114, USA

## Abstract

**Introduction:**

Heparin-induced thrombocytopenia (HIT) is described as a decrease in platelet count associated with heparin administration and is an immune-mediated adverse drug reaction that can cause both arterial and venous thromboses. It can be a life-threatening complication of heparin exposure. Little data concerning incidence, predisposing factors, or outcome in critically ill surgical patients are available.

**Methods:**

All critically ill, postoperative patients admitted between January 1, 2000, and December 31, 2001, to a surgical intensive care unit (SICU) who tested positive by an enzyme-linked immunosorbent assay for the HIT antibody (HPIA; Diagnostica Stago, Inc., Parsippany, NJ, USA) were identified. Patient risk factors and outcomes were abstracted retrospectively from the medical record and compared with those from control patients matched for age, gender, diagnosis, severity of illness, and date of SICU admission.

**Results:**

Two hundred and ten patients out of 2,046 patients (10%) admitted to the SICU had HIT assays performed. Nineteen patients (0.9% of admissions; 9% of tested individuals) had positive tests. HIT-antibody-positive patients, compared with 19 matched controls, had an increased risk of death or major thrombotic complications (37% versus 10%; *P *< 0.05) and prolonged length of intensive care unit (ICU) stay (20 days versus 10 days; *P *< 0.05). Exposure to heparin via intravascular flushes alone was sufficient to generate HIT antibodies in 12 of 19 (63%) patients. Five patients received platelet transfusions after the diagnosis of HIT was known; four of these patients died.

**Conclusion:**

Heparin flushes were the most common cause of HIT in this study. HIT-antibody-positive patients had an increased risk of death or major complications and a prolonged length of ICU stay. Platelet transfusions often were administered despite a positive HIT test result and were associated with a high mortality rate. Treatment algorithms that minimize exposure to heparin and contraindicate platelet transfusions merit further study.

## Introduction

Heparin-induced thrombocytopenia (HIT) (type II) is a potentially life-threatening complication of heparin exposure. It is defined as a decrease in platelet count associated with heparin administration in patients with detectable HIT antibodies. It is one of the most common immune-mediated adverse drug reactions, with 1% to 3% of all patients receiving unfractionated heparin at therapeutic levels (4 to 14 days) developing HIT [1]. It may develop sooner in patients who have previously received heparin [2]. The platelet count will usually decrease below 100 × 10^9 ^per liter or 50% of the baseline value and then return to normal after the heparin is discontinued, usually within 7 to 10 days [3]. Many patients develop arterial or venous thrombosis. Arterial thrombosis most commonly affects the cerebral or large lower limb vessels, presenting as thrombotic stroke or acute limb ischemia. Venous thrombotic complications, in particular proximal deep vein thrombosis and pulmonary embolism, are common sequelae of HIT [2].

Extensive literature exists documenting the prevalence and clinical significance of HIT in hospitalized populations [1-5]. Seroconversion is extremely common (51% of patients) after cardiac bypass surgery, but the occurrence of thrombocytopenia and thrombotic complications is relatively uncommon [6-8]. Among critically ill surgical patients, in whom exposure to heparin is commonplace, there are limited data concerning the incidence, risk factors, and outcome of HIT. What has been demonstrated in the hospitalized population is a higher incidence of HIT in surgical patients than in medical patients and that heparin administered intravenously in large doses and for an extended period increases the risk of HIT [9,10]. Case studies have also demonstrated the probability of developing HIT with minimal heparin exposure via intravascular flushes to maintain patency of indwelling arterial or venous catheters [11-15]. In the current study, the outcomes of mortality, morbidity, length of stay, and platelet transfusions were measured for frequency among HIT patients and control patients who had undergone surgery and were exposed to heparin in the surgical intensive care unit (SICU).

## Materials and methods

The study was approved by our Institutional Review Board. Our hospital's laboratory was used to identify patients who tested positive for HIT by commercial enzyme-linked immunosorbent assay (ELISA) (HPIA; Diagnostica Stago, Inc., Parsippany, NJ, USA) based on a threshold optical density (OD) measurement that is specific for each test day. All SICU patients who tested positive for HIT between January 1, 2000, and December 31, 2001, were enrolled in the study.

To further support the diagnosis of HIT, an objective clinical probability scoring system was used to retrospectively calculate the likelihood of HIT according to criteria of '4 T' score developed by the International Society on Thrombosis and Haemostasis SSC (Scientific and Standardisation Committee) Subcommittee on Platelet Immunology [16,17]. We used a modified version by Lo *et al*. [18] in which three clinical probability scores were divided into high (6 to 8 points), intermediate (4 to 5 points), and low (0 to 3 points) groups based on the final score (Table 1).

All patients had been admitted to the SICU for reasons other than HIT. The clinical course, risk factors, and outcomes of mortality, length of stay, morbidity, and occurrence of platelet transfusions were compared with data derived from control patients. We matched control patients by gender, similar admitting diagnosis, age (within 10 years of the HIT-positive [HIT^+^] patient), and severity of illness based on Acute Physiology and Chronic Health Evaluation (APACHE) II scores taken on the day of admission to the SICU. Each control patient resided in the unit within the same time period as the study patient and was thrombocytopenic.

We hypothesized that heparin exposure, extensive surgical procedures, large blood loss during surgery, trauma, and bacteremia might be possible risk factors that influence the incidence or outcome of HIT. Through chart review, we compared these risk factors and the clinical course in both study and control patients and related them to the occurrence of HIT.

## Results and Discussion

During the study period, 2,046 patients were admitted to the SICU. Of these patients, 210 (10%) had HIT assays performed because of clinical suspicion of HIT. Nineteen patients (9% of patients tested; 0.9% of admissions) had positive tests, supporting the clinical diagnosis of HIT. These 19 HIT^+ ^patients were well matched to 19 control patients with respect to gender, age, admitting diagnosis, and severity of illness based on APACHE II scores obtained on the day of admission to the SICU. Their clinical characteristics are listed in Table 2. The majority of the patients tested for HIT antibodies had a high positive ELISA OD titer measurement, whereas the four controls had a negative titer (Table 3).

All controls except two were thrombocytopenic while in the SICU. The two controls who were not thrombocytopenic did show a decrease in platelet count of 38% (from 480 to 318) in one and 7% (from 242 to 226) in the other. Only four controls were tested for HIT antibodies, and the results were negative. When the '4 T' scoring system of Lo *et al*. was applied to our HIT patients, four (21%) HIT patients were in the category of 'high' probability and 15 (79%) were in the 'intermediate' category. The 'high' probability scores supported the clinical diagnosis of HIT. These patients had a greater than 50% decrease in platelet count between 5 and 10 days after heparin exposure, and all developed a thrombus. The 'intermediate' scores strongly suggested the diagnosis of HIT, but according to the '4 T' scoring system this cannot be completely definitive, because these patients were also bacteremic, receiving antibiotics, had multiple system organ failure, and/or experienced postoperative bleeding. These patients had a greater than 50% decrease in platelet count between five and ten days after heparin exposure. None of these patients developed a thrombus, but we did observe platelet counts returning to normal within ten days after cessation of heparin, supporting the diagnosis of HIT. When the same scoring system was applied to the 17 controls who were thrombocytopenic, they fell into the 'low' probability category, as did the two controls without thrombocytopenia. Seventeen controls had a platelet count decrease between 25% and 50% and an onset of three to ten days after heparin exposure. The controls matched in this study had undergone extensive procedures and had large blood losses during surgery; this may be the cause of the low platelet counts. One control died of coagulopathy after extensive trauma and several surgical procedures.

Heparin exposure before diagnosis was limited to intravascular 'flush' solutions in 12 of 19 HIT^+ ^patients (and in 11 of 19 controls). Of these 12 HIT^+ ^patients, seven had some previous exposure to heparin (four prior to the SICU admission and three from another hospitalization). The remaining five had no known previous exposure to heparin and received only intravascular flush solutions. The standard flush solution is 1,000 units of heparin in 1,000 ml of 0.45% normal saline. Five units of heparin are delivered every hour to flush the intravascular line. An arterial catheter would receive 120 units in the course of a 24-hour period. A central venous catheter would receive 240 units and a pulmonary artery catheter would receive 360 units in a 24-hour period. The average lengths of stay in the SICU were 14 days for study subjects and 6 days for the controls. There was no association between HIT and extensive surgical procedures, large blood loss or trauma.

Platelet count decreased by 50% or more compared with the count upon admission to the intensive care unit (ICU) in all 19 patients who tested positive for the HIT antibody. Eleven of the 19 HIT^+ ^patients had a typical onset of thrombocytopenia occurring four days or more from the initial exposure to heparin in the SICU. The remaining eight of the 19 HIT^+ ^patients had a rapid onset of less than four days from the initial exposure to heparin in the SICU. Normalization of platelet count within ten days after cessation of heparin was observed in 14 of the HIT^+ ^patients. The remaining five patients expired before their platelet counts could return to normal.

### Statistical analysis

We performed an analysis of demographic and clinical factors and outcome. Normally distributed continuous data are presented as mean ± standard deviation unless otherwise indicated. Nonparametric data are presented as median ± 95% confidence interval (CI). Bivariate analyses were performed to evaluate the association between outcome variables and each of the dependent variables. For continuous data, comparisons between groups were made by unpaired *t *tests. Comparisons of discrete dependent and independent variables were performed with the Fisher's exact test, and odds ratios and 95% CIs were calculated for 2 × 2 comparisons. Survival analysis was performed by the Kaplan-Meier method. Results were considered statistically significant at a two-tailed *P *< 0.05. NCSS 97 software (NCSS, Kaysville, UT, USA) was used for all analyses.

### Outcomes

The presence of HIT was associated with an increased risk of death (relative risk 6.0, 95% CI 0.8 to 131.5; *P *= 0.09) (Table 4). The length of stay in the ICU was doubled for HIT^+ ^patients compared with controls. Among patients surviving to hospital discharge, HIT patients had fewer ICU-free days compared with control patients during the first 28 days. Overall hospital length of stay was increased in patients with HIT compared with controls.

Four of 19 HIT^+ ^patients had major thromboses (femoral vein thrombus, left atrial thrombus, ischemic stroke, and subclavian vein thrombus). One control patient had a deep venous thrombosis. The presence of HIT was associated with an increased risk of a combined endpoint of the occurrence of death or major thrombotic complications (relative risk 4.5, 95% CI 1.1 to 29; *P *< 0.05).

Twelve HIT^+ ^patients (versus five controls) received platelet transfusions either during surgery or postoperatively in the SICU. Five HIT^+ ^patients received platelet transfusions after the diagnosis of HIT was known; four of these patients died. Two of the four patients who died were actively bleeding, which influenced the decision to give platelets. A clear indication for platelet transfusion could not be determined from the medical record in the other two cases; both had postoperative complications of sepsis and multiple system organ failure, and thus we cannot attribute their deaths entirely to HIT and platelet transfusions. Four control patients were tested for HIT antibodies, and the results were negative. All controls except two were thrombocytopenic while in the SICU, and five received platelet transfusions. Of these five, one died from coagulopathy and the others did not display any further complications.

Platelet transfusions were administered despite the HIT status being suspected and confirmed by positive test results. For several reasons, platelet transfusions are not recommended in the setting of HIT. First, thrombocytopenia is due to accelerated consumption and not decreased production. Second, HIT is known to be associated with platelet-based thrombotic complications. Third, the platelet count is not predictive of the risk of hemorrhage in patients with HIT; and, fourth, bleeding complications are rare [19-22]. Nevertheless, in some cases, our clinicians appeared to react to the low platelet count by administering platelet transfusions.

## Conclusion

The occurrence of HIT after exposure to only small quantities of heparin has been reported [23-25], but the fact that the majority of HIT was due to flushes alone was unexpected, as this was previously believed to be an uncommon occurrence. Clinicians should consider the occurrence of HIT in patients who experience a platelet count decrease while receiving heparin flushes. When thrombocytopenia occurs, heparin from flushes may not be suspected initially as a cause of thrombocytopenia and may go unnoticed. This oversight may postpone the treatment of HIT and could result in serious morbidity and mortality. Heparin should be discontinued if HIT is suspected, with or without evidence of thrombosis, and alternative anticoagulation should be started [26,27].

Limitations in this retrospective case-control study were encountered in extracting accurate data concerning previous heparin exposure from the medical record. Some of the patients in this study were referred to our institution from other hospitals. Thus, we did not have access to all previous medical records and, due to regulations of the Health Insurance Portability and Accountability Act of 1996, could not request the charts retrospectively. Several of the patients in this study were trauma patients admitted through the emergency room. Their complete medical history prior to the emergent admission was unknown. We recorded previous heparin exposure in our data collection only when it was clearly documented in the medical records available to us. Another limitation is that control patients did not all receive testing for HIT antibodies even though they all had a decrease in platelet count and 17 displayed thrombocytopenia. Only four control patients had HIT ELISAs performed while in the SICU. Patients were otherwise matched for age, gender, admitting diagnosis, date of admission, and severity of illness based on APACHE II score, however, suggesting strongly that the outcome differences that we observed were due to the presence of HIT. Of course, the results of this study cannot be generalized to other populations of patients in other intensive care settings.

As this was a retrospective study, we had only the ELISA test available to detect HIT antibodies, and this test has a high sensitivity and a relatively low specificity [28]. We were unable retrospectively to perform another test that has higher specificity, such as the serotonin release assay or the heparin platelet aggregation test, given that our hospital does not store blood specimens. Such tests would possibly have provided further support of the diagnosis of HIT in our cases. The clinical probability scoring system of '4 T' confirmed high probability in four cases and clinically supported the diagnosis of HIT. Fifteen cases had scores of 'intermediate,' which strongly suggests HIT, but other factors such as bacteremia, antibiotics, multiple organ system failure, and postoperative bleeding may have contributed to the diagnosis. Retrospectively, if we had been able to perform another test with high specificity (serotonin release assay) on these 15 cases to distinguish pathogenic immunoglobulin G (IgG) class antibodies from nonpathogenic IgM and IgA class antibodies, we would have additional verification of the diagnosis of HIT.

This study suggests further investigation, including randomized clinical trials of alternatives to standard unfractionated heparin as intravascular flush solutions in critically ill patients. Treatment algorithms that would minimize exposure to heparin (including flush solutions) and algorithms that would prevent unnecessary platelet transfusions also merit further study. As a result of this review, we identified two process variables that could be improved: identification of the patient as HIT^+ ^and prevention of platelet transfusions among HIT^+ ^patients.

## Key messages

• There are little data available in the literature related to incidence, predisposing factors, or outcomes in critically ill surgical patients diagnosed with HIT.

• This retrospective observational study examined risk factors that might predispose surgical patients to HIT.

• There was no association between HIT and extensive surgical procedures, large blood loss, or trauma.

• The study did discover that minimal exposure to heparin via intravascular flushes in the majority of the subjects was sufficient to generate HIT, and this was previously believed to be an uncommon occurrence.

• Lastly, the study showed that platelet transfusions were administered in five cases despite the diagnosis of HIT being known, which suggests the need for treatment algorithms that would prevent unnecessary platelet transfusions.

## Abbreviations

APACHE = Acute Physiology and Chronic Health Evaluation; CI = confidence interval; ELISA = enzyme-linked immunosorbent assay; HIT = heparin-induced thrombocytopenia; ICU = intensive care unit; Ig = immunoglobulin; OD = optical density; SICU = surgical intensive care unit.

## Competing interests

For each author listed on this manuscript, there is no personal or financial support or author involvement with organizations with financial interest in the subject matter and no conflict of interest exists. The authors declare that they have no competing interests.

## Authors' contributions

EG carried out the literature search, chart review, data collection, and drafted and revised the manuscript. All authors participated in the design of the study. WH performed the statistical analysis, provided supervision to the group, and helped to draft the manuscript. KB conceived of the study, participated in its design and coordination, and helped to draft the manuscript. EV assisted with acquisition of data (ELISA results) and interpretation of data and was involved in drafting and revising the manuscript. All authors read and approved the final manuscript.

## Figures and Tables

**Table 1:** Pretest scoring system for HIT: the 4 Ts

4 Ts	2 points	1 point	0 points
Thrombocytopenia	Platelet count >50% and platelet nadir = 20*	Platelet count decrease 30% to 50% or platelet nadir 10 to 19	Platelet count decrease < 30% or platelet nadir < 10
Timing of platelet count decrease	Clear onset between days 5 and 10 or decrease = 1 day (prior heparin exposure within 30 days)	Consistent with decrease between days 5 and 10, but not clear (for example, missing platelet counts); onset after day 10; or decrease = 1 day (prior heparin exposure 30 to 100 days before)	Platelet count decrease < 4 days without recent exposure
Thrombosis or other sequelae	New thrombosis (confirmed); skin necrosis; acute systemic reaction after intravenous unfractionated heparin bolus	Progressive or recurrent thrombosis; non-necrotizing (erythematous) skin lesions; suspected thrombosis (not proven)**	None
Other causes for thrombocytopenia	None apparent	Possible++	Definite++

The clinical probability scores are high (6 to 8 points), intermediate (4 to 5 points), and low (0 to 3 points) [18]. HIT, heparin-induced thrombocytopenia.* Platelet count fall >50% or nadir 20–100. **Suspected thrombosis(not proven) was not included as a criterion. ++ Determination of whether the presense of another apparent cause of thrombocytopenia was "possible" or "definite" was at the discretion of the investigator.

**Table 2:** Clinical characteristics of patients

Characteristics	HIT^+ ^(*n *= 19)	Control (*n *= 19)
Demographics		
Age in years (range 21 to 86), mean ± SD	61 ± 19	61 ± 20
Male, *n *(percentage)	12 (63)	12 (63)
Female, *n *(percentage)	7 (37)	7 (37)
Clinical		
Diagnosis, *n *(percentage)		
Gastrointestinal	7 (37)	6 (32)
Trauma	6 (32)	6 (32)
Vascular	3 (16)	3 (16)
Other	3 (16)	4 (22)
Bacteremias, *n *(percentage)	7 (37)	3 (16)
APACHE II score, mean ± SD	24 ± 6	23 ± 8
Heparin exposure		
Intravascular flush, *n *(percentage)	19 (100)	18 (95)
Subcutaneous	4 (21)	6 (32)
Intravenous	3 (16)	2 (11)
Previous exposure	13 (68)	5 (26)

APACHE, Acute Physiology and Chronic Health Evaluation; HIT^+^, heparin-induced thrombocytopenia-positive; SD, standard deviation.

**Table 3:** ELISA test results compared with morbidity and mortality

Subject	ELISA OD titer	ELISA OD cutoff	Positive blood cultures in SICU^a^	Platelets given after HIT^+a^	Thrombus^a^		Cause of death	Control	ELISA OD titer	ELISA OD cutoff	Positive blood cultures in SICU^a^	Platelets given^a^	Thrombus^a^		Cause of death
													
					Venous	Arterial							Venous	Arterial	
1	1.293	0.541		Yes			Sepsis/MOSF	1				Yes			Trauma/Coagulopathy
2	1.388	0.580	Yes	Yes			Sepsis/MOSF	2				Yes			
3	2.502	0.578						3	0.150	0.537					
4	3.556	0.712	Yes	Yes	Yes		Cardiac arrest	4							
5	0.607	0.285		Yes		Yes		5							
6	2.107	0.380						6							
7	1.764	0.533						7			Yes	Yes			
8	3.665	0.393						8							
9	0.606	0.446						9							
10	2.938	0.776	Yes					10	0.042	0.692		Yes			
11	1.082	0.774					Sepsis/Liver failure	11	0.032	0.774	Yes				
12	2.141	0.600	Yes		Yes			12	0.082	0.506					
13	1.45	1.39	Yes					13							
14	1.25	1.11		Yes			Sepsis/MOSF	14			Yes				
15	1.349	0.784						15					Yes		
16	0.726	0.68	Yes				Sepsis/Pancreatitis	16							
17	0.910	0.710				Yes		17							
18	0.656	0.539						18			Yes	Yes			
19	0.81	0.61	Yes					19							

^a^An empty cell indicates 'No'. ELISA, enzyme-linked immunosorbent assay; HIT^+^, heparin-induced thrombocytopenia-positive; MOSF, multiple organ system failure; OD cutoff, optical density results above this value are positive (measurement specific for that day of testing); OD titer, optical density of patient result; SICU, surgical intensive care unit.

**Table 4:** Patient outcomes

Outcomes	HIT^+ ^(*n *= 19)	Control (*n *= 19)	*P *value
Hospital deaths, *n *(percentage)	6 (32)	1 (5)	0.09
Thrombotic event, *n *(percentage)	4 (19)	1 (5)	< 0.05
^a^Death or thrombotic event,*n *(percentage)	9 (47)	2 (10)	< 0.05
ICU LOS in days, mean ± SD	20 ± 15	10 ± 9	< 0.02
Median	14	6	
Interquartile range	8 to 29	3 to 11	
Hospital LOS in days, mean ± SD	33 ± 20	28 ± 25	< 0.02
Median	40	15	
Interquartile range	25 to 46	10 to 34	
ICU-free days, mean ± SD	7 ± 0	17 ± 21	< 0.0002

^a^Combined endpoint of death or thrombotic event. ICU-free days (during the first 28 days of hospital stay). HIT^+^, heparin-induced thrombocytopenia-positive; ICU, intensive care unit; LOS, length of stay; SD, standard deviation.
